# Structure Basis for Chloride Recognition and Transport by Human Kidney Brain Anion Transporter (KBAT)

**DOI:** 10.3390/biom16101384

**Published:** 2026-09-23

**Authors:** Yurou Wang, Tengyu Xie, Songyue Wu, Anxin Wang, Wentao Cheng, Jing Huang, Renhong Yan, Ximin Chi

**Affiliations:** 1 State Key Laboratory of Cellular Stress Biology, School of Life Sciences, Faculty of Medicine and Life Sciences, Xiamen University, Xiamen 361102, China; wangyrou@stu.xmu.edu.cn (Y.W.); 21620251153549@stu.xmu.edu.cn (A.W.); chengwentao@stu.xmu.edu.cn (W.C.); 2 College of Information Engineering, Zhejiang University of Technology, Hangzhou 310023, China; xietengyu@westlake.edu.cn; 3 State Key Laboratory of Gene Expression, School of Life Sciences, Westlake University, Hangzhou 310030, China; huangjing@westlake.edu.cn; 4 Department of Biochemistry, SUSTech Homeostatic Medicine Institute, School of Medicine, Key University Laboratory of Metabolism and Health of Guangdong, Institute for Biological Electron Microscopy, Southern University of Science and Technology, Shenzhen 518055, China; 12233037@mail.sustech.edu.cn

**Keywords:** kidney brain anion transporter (KBAT), protonation-dependent Cl^−^ recruitment, transport conformational switch

## Abstract

SLC26A11, also known as the kidney and brain anion transporter (KBAT), mediates the transport of multiple anionic substrates across the plasma membrane. It is highly enriched in cerebellar Purkinje cells, where loss of KBAT results in hyperpolarized membrane potentials and increased intrinsic firing frequency, correlating with locomotor dysfunction. Despite its physiological significance, the molecular basis of substrate recognition and transport by KBAT remains elusive. Here, we present the structure of full-length human KBAT in an inward-facing conformation with chloride ions (Cl^−^) bound. Sequence analysis of the substrate-binding pockets uncovers the important residue E320 in KBAT. Protonation of E320 abolishes its interaction with S363 and correlates with Cl^−^ binding in the canonical substrate pocket, as substantiated by molecular dynamics (MD) simulations. Quantitative MD contact analysis delineates the rearrangements of residue interactions underlying the alternative access transport, consistent with an elevator-like transport mechanism. The cross-arranged transmembrane helices of the Gate and Core domains require minimal conformational rearrangement to achieve alternating access to the membrane. Collectively, our study provides a critical foundation for comprehensively understanding the recognition and transport mechanisms of human KBAT, with potential implications for developing therapies for neuronal swelling.

## 1. Introduction

Solute carrier protein family 26 consists of 11 genes in human, with 10 proteins encoded. SLC26 proteins are capable of multiple anion substrate transport, including sulfate, chloride, iodide, bicarbonate, oxalate, thiocyanate, et cetera [1,2,3,4,5]. The regulation of major anion transport by SLC26 transporters is implicated in diverse physiological processes, including the maintenance of intestinal epithelial integrity, thyroid hormone biosynthesis, sulfate uptake in chondrocytes, salt homeostasis in the kidney, and electromotility of the cochlea. Collectively, these critical roles render SLC26s a focal point of research interest [1,3,6,7,8,9].

KBAT, also known as the kidney and brain anion transporter, is ubiquitously expressed across multiple organs, with the highest expression levels observed in the placenta, kidney, and brain [10]. In the kidney, KBAT co-localizes with H^+^-ATPase on the apical membrane of acid-secretory A-type intercalated cells within the collecting duct, where it facilitates acid secretions [11]. In the brain, KBAT is predominantly expressed in cerebellar Purkinje cells and cooperates with vacuolar H^+^-ATPase to modulate intracellular pH homeostasis [12]. In these KBAT-enriched cerebellar Purkinje cells, deletion of KBAT leads to diminished Cl^−^ influx, hyperpolarized membrane potentials, and elevated intrinsic firing frequency, supporting its functional role as a chloride accumulator. KBAT knockout mice exhibit an increased frequency of small steps in the Erasmus Ladder test, a behavioral paradigm considered indicative of locomotor impairment [13]. Cytotoxic edema resulting from brain trauma or cerebral infarction is characterized by neuronal swelling, during which depolarization and sodium influx trigger secondary, KBAT-dependent chloride influx, ultimately leading to neuronal death [14]. In a rat stroke model, KBAT mRNA and protein levels were elevated near the infarct core, contributing to neural swelling [15]. Notably, pharmacological inhibition of KBAT attenuates neuronal swelling and cell death, thereby presenting a promising therapeutic strategy for stroke [14,15]. Emerging research has further expanded the recognized involvement of KBAT in diverse physiological and pathological processes. The KBAT G566R mutation, identified in a family with thyroid cancer, has been characterized as a cancer predisposition gene [16]. In mouse pyramidal neurons, static magnetic field exposure activates a DIDS-sensitive chloride channel—pharmacologically consistent with KBAT—and transiently reduces neuronal excitability [17]. Additionally, KBAT has been identified as a potential biomarker among immune-related genes upregulated following imatinib treatment in patients with chronic myeloid leukemia [18].

KBAT has been reported to exhibit diverse transport functionalities, reflecting its multifaceted role in ion homeostasis. When heterologously expressed in HEK293T cells, KBAT induces a constitutive chloride conductance [12]. Transient expression studies in COS-7 cells have further demonstrated its capacity for Cl^−^/HCO_3_^−^ exchange activity, implicating a potential role in bicarbonate-dependent pH buffering [11]. Electrophysiological analyses have demonstrated that KBAT possesses voltage-gated Cl^−^ channel activity, with its conductance being modulated by alterations in membrane potential. Loss of KBAT function results in neuronal swelling, followed by cell death [14]. Beyond Cl^−^, KBAT is capable of conducting additional halide ions, including bromide (Br^−^) and iodide (I^−^), when heterologously expressed in HEK293 cells [13]. Furthermore, functional expression in SF9 cells identifies KBAT as a sodium-independent sulfate transporter, with activity inhibited by the anion-exchange blocker DIDS [10], reinforcing its classification within SLC26 family of multifunctional anion exchangers.

KBAT exhibits limited sequence conservation relative to other SLC26 family proteins, suggesting the existence of unique structural features. Here, we report the cryo-EM structure of human KBAT in inward state. Protonation of E320 enables stable Cl^−^ binding within the canonical substrate site nestled between half-helices TM3 and TM10. Through quantitative MD contact occupancy analysis, we further characterize how residue interactions are reorganized between the inward- and outward-facing conformations, lending support to an elevator-type transport model for KBAT. These findings shed light on the mechanistic basis of Cl^−^ transport by KBAT and highlight this transporter as a promising therapeutic target for interventions aimed at alleviating neuronal swelling.

## 2. Materials and Methods

### 2.1. Cloning, Expression, and Purification of Human KBAT

The full-length protein-coding DNA segment for human *SLC26A11* (UniProt ID: Q86WA9) was amplified from a cDNA library and subcloned into the pSQ vector. The Flag tag and Strep tag was fused to the C-terminus of KBAT. HEK 293F cells were cultured in SMM 293-TII medium under 5% CO_2_, 37 °C, 130 rpm (ZCZY-BS8, ZHICHU, Shanghai, China). Transfection was carried out when the cells reached a density of ~2.0 × 10^6^ cells/mL. Approximately 1.5 mg plasmids and 4 mg polyethylenimine (PEI, MW40000, LABLEAD, Beijing, China) were applied to each liter of cells. After 48 h, cells were harvested in lysis buffer containing 25 mM Tris, pH 8.0, and 150 mM NaCl by centrifugation at 2000× *g* for 10 min. Then the cells were lysed in 1.5% n-dodecyl-β-D-maltopyranoside (DDM) with 0.2 mM phenylmethylsulfonyl fluoride (PMSF) at 4 °C for 2 h. After centrifugation at ~16,000× *g* for 50 min, the supernatant was applied to anti-Flag G1 affinity resin at 4 °C. The resin was washed with 0.01% glyco-diosgenin (GDN) and eluted in the same buffer with an additional 0.25 mg/mL Flag peptide. Then the protein was further purified by size exclusion chromatography (Superose 6 Increase 10/300 GL, purchased from Cytiva, China office, Shanghai, China; manufacturer: Cytiva, Little Chalfont, Buckinghamshire, UK) in buffer (25 mM Tris pH 8.0, 150 mM NaCl, 0.01% GDN). The peak fraction was analyzed by SDS-PAGE and concentrated for cryo-EM sample preparation.

### 2.2. Cryo-EM Sample Preparation and Data Acquisition

The purified KBAT sample was concentrated to approximately 12 mg/mL before being applied to glow-discharged holey carbon grids (Quantifoil Cu R1.2/1.3, Quantifoil Micro Tools GmbH, Großlöbichau, Germany). Aliquots of the protein sample (3.5 μL) were placed on the grids. The grids were then blotted for 3.5 s and flash-frozen in liquid ethane cooled by liquid nitrogen using the Vitrobot (Mark IV, Thermo Fisher Scientific, Waltham, MA, USA). The grids were then transferred to a Titan Krios G3 transmission electron microscope (Thermo Fisher Scientific, Eindhoven, The Netherlands) operating at 300 kV, equipped with a Gatan K3 Summit detector (Gatan, Inc., Pleasanton, CA, USA) and GIF Quantum energy filter (Gatan, Inc., Pleasanton, CA, USA). Movie stacks were automatically collected using EPU software (version 3.12) with a slit width of 20 eV on the energy filter. The defocus range was set from −0.8 µm to −1.5 µm in super-resolution mode at a nominal magnification of 105,000×. Each stack was exposed for 1.71 s, with an exposure time of 0.053 s per frame, resulting in a total of 32 frames per stack. The total dose rate for each stack was approximately 50 e^−^/Å^2^. Subsequently, the stacks were motion-corrected using MotionCor 2 [19]. After motion correction, the movie stacks were binned 2-fold, resulting in a pixel size of 0.827 Å/pixel. Dose weighting was then applied to the data. The defocus values were estimated with Gctf [20].

### 2.3. Cryo-EM Data Processing and Model Building

The Cryo-EM structures of KBAT were solved in cryoSPARC 4.6.2 [21]. Particles were automatically picked using cryoSPARC 4.6.2. After 2D classification, the micrographs with good particles were selected, and these particles were subjected to several cycles of 2D classification, ab initio reconstruction, and multiple cycles of heterogeneous refinement without symmetry. The good particles were selected and subjected to local CTF refinement with C1 symmetry, non-uniform refinement, and local refinement, resulting in the 3D reconstruction for the whole structures. The resolution was estimated with the gold-standard Fourier shell correlation 0.143 criterion with high-resolution noise substitution. Refer to Figure S1 and Table S1 for details of data collection and processing.

Molecular model of human KBAT predicted by AlphaFold 3 (AF3, AF-Q86WA9-F1) [22] was fit to the cryo-EM map by Chimera (version 1.17) as the initial structure model [23]. After initial model fitting, models were manually modified using COOT (version 0.9.8.92) [24] and refined using PHENIX (version 1.20.1-4487) [25].

### 2.4. Molecular Dynamics Simulation

We constructed six molecular dynamics (MD) simulation systems to investigate KBAT. Four systems are initiated from the experimental structue (exp), spanning combinations of bound (holo) vs. unbound (apo) chloride ion and charged (E320^−^) vs. protonated (E320^H^) E320. The former condition aims to the stability of the bound Cl^−^ and its interaction with protein, while the latter one examines the potential role of E320 as a proton sensor regulating transport. The last two systems were initiated with the structure predicted by AF3 (AF3) with different protonation states of E320, aiming to evaluate its plausibility and compare it with the experimental structure. Only the monomeric form of KBAT was simulated, which is sufficient for addressing the structural questions here. As for the unresolved segments (residues: 1–27 and 223–234) in the experimental structure, the N-terminal segment was omitted in all systems, while the later segment was patched from the predicted structure. All systems were constructed using CHARMM-GUI [26,27] with the CHARMM36m [28] force field for proteins. The structures were embedded into a 1-palmitoyl-2-oleoyl-sn-glycero-3-phosphocholine (POPC) lipid bilayer. Protonation states were assigned at pH 7.0 using PROPKA3.1 [29], except for E320. Systems were solvated with TIP3P water [30] and neutralized with 0.15 M NaCl. The simulation boxes measured approximately 112 × 125 × 143 Å^3^ containing ~198,000 atoms. Energy minimization was carried out using steepest-descent and adopted-basis Newton–Raphson algorithms (50 steps each). Solvent relaxation was performed under NVT conditions for 5 ps, with a restraint force constant of 400.0 kJ/mol/nm^2^ on protein backbone and small-molecule-heavy atoms and that of 40.0 kJ/mol/nm^2^ on protein side-chain atoms. Production simulations were conducted using OpenMM [31] at 310 K and 1 bar with a 2 fs timestep. Long-range electrostatics were computed using the particle-mesh Ewald method [32] with a 12 Å real-space cutoff, and Lennard–Jones interactions were smoothed between 10 Å and 12 Å. Hydrogen-involving bonds were constrained with SHAKE [33]. Each simulation was run for 100 ns and repeated three times with different random seeds. For each simulation, one frame was saved every 100 ps, and 1000 frames were generated for analysis. Trajectory analyses, including RMSD and binding analysis, were performed using MDAnalysis (version 2.3.1) [34].

## 3. Results

### 3.1. Structural Architecture Human KBAT

To dissolve the molecular architecture and transport mechanism of KBAT, human *SLC26A11* was heterologously expressed in HEK293F cells, and then the purified KBAT in GDN was subjected to Cryo-EM. 3D reconstruction was obtained after data processing, achieving an overall resolution of 3.5 Å and a local resolution about 2.0 Å, thus enabling the atomic model building of most regions of the protein (Figure S1). Detailed protein purification, cryo-EM data collection, and processing procedures can be found in the Section 2. (Figure S1; Table S1).

KBAT is arranged as a domain-swapped homodimer, as the other mammalian SLC26 protein structures [35,36,37]. The sulfate transporter anti-sigma (STAS) domain is located on the cytosolic side and placed beneath the transmembrane domain (TMD) from the other protomer (Figure 1A). Multiple lipid-like molecules are presented around the transmembrane (TM) helices (Figure 1B). The TMD is constructed from 14 transmembrane (TM) helices and can be divided into Core and Gate domains. The Core domain is composed of TM1–TM4 and TM8–TM11, while the Gate domain constitutes TM5–TM7 and TM12–TM14. TM1–7 and TM8–14 form a pair of pseudosymmetry units that interwind with each other, whereas TM5b appears additionally. TM3 and TM10 are half-unwound transmembrane helixes (Figure 1C,D). The loop connecting TM7 and TM8 (EL78) in the Core domain is extended in KBAT, which is stabilized by hydrophobic interaction between V290 and I297, compared with the SLC26A9 structure (Figure 1D,E). In the Gate domain, a special bending is presented near the TM7 N-terminus (Figure 1D). The STAS domain can be continuously built in the model with three parallelly located β strands surrounded by four α-helices (Figure 1D). Compared with the STAS domain of Prestin, the connection loops between α1 and β2 are shortened in KBAT (Figure 1F).

There are two dimerization interfaces that exist in the structure model of KBAT. One of the dimerization interfaces lies in the STAS domain, which is composed of hydrophilic interactions between H520 and Q552′. The continuous cryo-EM map density also supports close interactions between C522 and H520 from different protomers (Figure 1G). A leucine zipper formed by L461, L464, and L465 from C terminal of TM14 from different protomers contributes to another dimerization interface. In addition, F148 from IL45, and F491 from the STAS domain (Loop β1-α1) further extend the hydrophobic core (Figure 1H).

### 3.2. Substrate Cl^−^ Recognition by KBAT

Partial unwinding of transmembrane helices TM3 and TM10 enables substrate coordination by mainchain chemical groups, constituting the canonical substrate-binding site in SLC26 transporters. A well-defined extra cryo-EM density is present at this site and was assigned to a chloride ion, in line with the high chloride concentration used throughout purification (Figure 2A). The chloride ion is mostly coordinated by side-chains of S363, Q65, and Y69, and mainchain amide nitrogen from F364, G365, A106, and T105 (Figure 2B). The locations of the substrate halide ions are highly conserved among SLC26 homologs, further supporting the assignment of Cl^−^ ion in this pocket (Figure 2C).

To further probe the viability of stable Cl occupancy at this site, molecular dynamics (MD) simulations were performed. After aligning the trajectories to the Gate domain of the initial structure, backbone RMSD calculations over the transmembrane domain showed that all systems reached a stable plateau within ~20 ns (mean RMSD ≈ 2.2 Å; Figure S2A). RMSF analysis also indicated low fluctuations in the core regions (Figure S2B), supporting the overall stability under simulation conditions. Throughout the simulations, the coordination pocket consistently retained a single Cl^−^ ion, indicating stable chloride binding at this locus (Figure 2D). These results support both the overall stability of the system and the stable binding of the chloride ion within this pocket. In addition, multiple positively charged residues are present at the cytosolic and extracellular openings (Figure 2E), which may facilitate the recruitment and passage of negatively charged substrate ions, including chloride and sulfate.

When sequences of different SLC26A11 orthologues are aligned, most residues involved in Cl^−^ coordination are conserved, highlighting the functional conservation (Figure 2F). Sequence alignment among SLC26 paralogues reveals subtle differences. Residues whose side chains participate in Cl^−^ coordination exhibit either strict sequence conservation (colored blue) or share some similar properties (colored green), whereas those that coordinate Cl^−^ via main-chain groups show low sequence conservation (colored gray, Figure 2G and Table S2). Strict sequence conservation is observed in Q65 and G103. Q65 provides an amide group that facilitates anion substrate coordination in KBAT. G103 contributes to maintaining the half-unwound secondary structure of transmembrane helix 3 (TM3). Y69 is consistently represented by aromatic residues in SLC26 transporters but not strictly conserved. A mutation of its counterpart in Prestin (F101Y) decreases salicylate inhibition [35], supporting that difference of tyrosine and phenylalanine will affect transport characteristics of SLC26s. I107 and F364 are not strictly conserved but retain hydrophobic properties, enabling them to provide mainchain coordination sites while facilitating the rapid passage of anion substrates. R366 remains predominantly positively charged. Residues that participate in ion coordination via their mainchains—namely P104, A106, and G362—are not conserved across the family (Figure 2G; Table S2).

T105 is a distinctive component of KBAT, whereas phenylalanine predominates at this position in other SLC26 transporters. In SLC26A7, this phenylalanine acts as a substrate selector, where substitution with alanine or the positively charged residue lysine facilitates chloride and iodide transport [38]. In KBAT, T105 provides a main-chain amide group for coordination, rendering its side chain less critical (Figure 2G; Table S2). There was research supporting that KBAT colocalized with V H^+^-ATPase in plasma membrane of Purkinje cells can regulate acid transport [12]. Although E320 does not directly participate in Cl^−^ coordination (Figure 2B), it links proton sensitivity and Cl^−^ transport in KBAT. The localized pKa of E320 reaches 8.69 [39], which indicates that E320 is protonated in the cryo-EM sample solution. Yet the protonation state of E320 cannot be defined through Cryo-EM density maps.

To examine whether this E320 rearrangement directly affects chloride binding, we performed all-atom MD simulations starting from the experimental structure in both apo (Figure S3A) and holo states (Figure S3B), with E320 modeled in either its unprotonated or protonated form. We tracked the trajectories and residence behavior of chloride ions across simulation replicas. In simulations initiated from the apo state with protonated E320, a chloride ion from the bulk solution spontaneously entered the binding cavity within the first 5 ns and remained stably coordinated for over 90 ns. In contrast, when E320 was charged, chloride ions only occasionally approached the pocket vestibule with substantial positional fluctuations, failing to establish stable occupancy (Figure 3A and Figure S3A). Moreover, in simulations initiated from the experimental Cl^−^-bound holo structure (Figure S3B), the bound ion spontaneously dissociated into the solvent in the charged E320 state (replica 2), whereas the protonated state preserved robust coordination across all replicas, with only an immediate site-exchange event observed.

Contact analysis suggests that S363 is the primary coordinating residue exhibiting the highest contact occupancy (88.0%, Table S3) in ‘exp/apo/E320^H^’ simulations. To understand the structural basis for this protonation-dependent binding, we quantified local conformational changes around E320. In the charged state, E320 maintains a tight interaction with S363 (67.4% contact occupancy). However, upon protonation, this contact occupancy drastically drops to 1.4% (Figure 3B), accompanied by an increase in the mean Cα–Cα distance between E320 and S363 from ~6.5 Å to ~8.1 Å (Figure 3C). Visual inspection of representative snapshots (Figure 3D) indicates that protonation reorients the E320 side chain away from S363. This conformational shift effectively exposes S363, thereby facilitating the recruitment and stable coordination of the chloride ion.

Taken together, our results point to a model in which protonation of E320 abrogates the interaction with S363 and concurrently potentiates Cl^−^ binding within the pocket. We thus propose that E320 protonation constitutes a molecular switch that modulates both substrate stability and transport function.

### 3.3. Conformational Change During Transport Cycle

Following the alignment strategy established in previous studies [35,37,40], the Gate domain was used as a reference to illustrate the relative movements of the Core domain during the elevator transport process. With the Gate domains aligned, the Core domain of KBAT exhibits high similarity to that of SLC26A9 (PDB: 7CH1) [36], yielding an overall RMSD of 1.089 Å, consistent with an inward-facing (IN) conformation (Figure S2C). The Core domain of KBAT is situated more proximally to the cytoplasmic side than that of Prestin (intermediate state, PDB: 7LGU), further supporting that KBAT adopts an inward-facing conformation. Meanwhile, TM1, TM10, and TM8 on the cytoplasmic side also exhibit modest lateral displacements. Moreover, relative to the outward-facing Pendrin (Outward, PDB ID: 7WLE) [37], the core domain of KBAT adopts a more widely separated conformation (Figure S2D).

The AF3-predicted structure of KBAT was captured in a distinct conformational state. When aligned to the Gate domain, the Core domain of the AF3 model exhibits a pronounced upward displacement relative to the experimental structure. This displacement is accompanied by lateral movement when the viewing angle is rotated by ~90° (Figure 4A). Compared with other SLC26 paralogs, the AF3-predicted outward-facing KBAT model shows high similarity to the outward-facing (OUT) structure of Pendrin (RMSD 1.301 Å), indicating that the AF3-predicted KBAT structure adopts an outward-facing conformation (Figure S2D).

To evaluate the physical plausibility of the predicted conformation before comparing structural differences, we performed all-atom MD simulations starting from the AF3-predicted structure with two protonated states of E320 (‘AF3/E320^−^’ and ‘AF3/E320^H^’). This MD system was analyzed alongside simulations initiated from the experimental structure. In the simulations, RMSD values plateaued within the first ~20 ns and remained stable thereafter, with a mean RMSD of 1.6 Å for simulations starting from the predicted structure, which is comparable to simulations initiated from the experimental structure (Figure S2A). In addition, the MD results show low residue-level fluctuations in the transmembrane helices, indicating that the predicted conformation is stable during the simulation (Figure S2B). Together, these results support the plausibility of the AF3-predicted structure and enable further analysis of the conformational differences between the predicted and experimental states.

When aligned to the Gate domain, the Core domain undergoes a pronounced displacement toward the extracellular side in the predicted structure. Surface charge distribution analysis revealed a basic pocket between TM3 and TM10 that would facilitate anion substrate coordination. As the Core domain moves outward, this basic pocket corresponding to the canonical Cl^−^ binding site shifts accordingly (Figure 4A). Multiple diseases related mutations can be mapped in KBAT structure, especially around Cl^−^ binding site, including TM10 and TM3 (Figure S2E,F).

The interaction networks between the Core and Gate domains are rearranged in the transport process. To quantitatively define interfacial interactions during simulation, we calculated residue-level contact occupancies along MD trajectories. We focused on contacts exhibiting substantial occupancy shifts (>40%) between two kinds of simulations (Table S4). This analysis found that 16 Gate residues systematically switch interaction partners in the Core domain (Figure 4B,C). For example, I411 in the Gate domain contacts A68 and Y69 of the Core domain in the IF state but shifts to contact V369 in the OUT state. This partner switching supports vertical movement of the Core domain during elevator-like sliding with respect to Gate domain.

Beyond partner switching, we identified conformation-specific contacts that exist exclusively in one state. Mapping the IN-specific interactions onto the KBAT TMD indicates that they are predominantly situated on the extracellular side of the chloride substrate (Figure 4D). Conversely, the OUT-specific interaction residues are largely confined to the cytosolic side (Figure 4E, Table S4). Surface analyses show that partial residues border the positively charged chloride-binding vestibule (Figure S4A,B). The disruption of these contacts exposes the vestibule, whereas their engagement in the opposing state forms a physical seal that closes the cavity. We then tracked the diffusion of multiple chloride ions along the membrane normal (z-axis) relative to the protein center (Figure S4C). In OUT conformational simulations, chloride ions frequently penetrated the extracellular vestibule but were completely excluded from the intracellular side. Conversely, in IN simulations, chloride ions sampled the intracellular vestibule, with no persistent entry from the extracellular solution. These observations indicate that the state-specific contacts alternately seal the vestibule, which is consistent with the alternating-access mechanism of KBAT.

As the Core domain moves up and down relative to the Gate domain, substrates residing in the coordination site of KBAT become accessible to either side of the membrane. This dynamic mechanism represents the elevator transport model adopted by the SLC26 family proteins. The tilting of TM5 and TM12 within the Gate domain minimizes the distance required for Core domain displacement, thereby shortening the overall translocation path. Substrate is exposed to extracellular side in outward conformation by residing in the up-right of TM12 and TM5. Core domain only needs to move a small distance toward cytosol before the substrate occupies the left-down position of TM5 and TM12 and gains access to cytosolic side (Figure 4F).

## 4. Discussion

KBAT exhibits diverse physiological functions predominantly in the kidneys and brain. It acts as a major chloride entry pathway in rat stroke model, with potential clinical appliance in neuronal swelling and stroke treatments [15]. In this study, we revealed the chloride ion substrate recognition mechanism of human KBAT. Protonation of E320 correlates with chloride coordination in the canonical substrate binding site. Quantitative MD contact analysis elucidate residue contact occupancy changes in IN and OUT conformations, placing it within the conserved elevator model adopted by other SLC26s.

KBAT has been consistently documented to exhibit chloride channel activity [12,14]. Cl^−^ binding in KBAT is orchestrated primarily by main-chain amide groups within the unwound regions of TM3 and TM10, while the globally positive-charged pocket may synergistically enhance anion recruitment. Residues whose side chains participate in Cl^−^ coordination exhibit either strict sequence conservation (colored blue) or share some similar properties (colored green), whereas those that coordinate Cl^−^ via main-chain groups show low sequence conservation (colored gray, Figure 2G). Unlike the sequence variability observed among paralogues, orthologues display notable conservation, especially at substrate-coordinating residues (Figure 2F). Even those residues that engage Cl^−^ solely via their main-chain amide groups—namely P104, A106, I107, G362, and G365—are preserved, underscoring their role in secondary structure maintenance. Furthermore, certain residues, including T105, S363, F364, and R366, utilize main-chain coordination while also helping to shape the local environment.

KBAT is well established to colocalize with H^+^-ATPase and regulate intracellular pH via Cl^−^/HCO_3_^−^ exchange in the kidney [11] and cerebellum [12]. H^+^-dependent Cl^−^ transport has been reported for guinea pig SLC26A11 [41]. Sulfate uptake through SLC26A11 was blocked by alkaline extracellular pH in bovine articular chondrocytes [41]. Collectively, KBAT transport activity correlates with pH regulation, implying the existence of a proton-sensing mechanism. As the sole acidic residues in KBAT substrate binding pocket, the existence of E320 suggests a mechanism for pH-dependent regulation in KBAT (Figure 3). The role of glutamate residue as a proton sensor was reported in human EAAT3 structures with endosomal localization, where protonation of E374 disrupts its interaction with R447, thereby enabling substrate loading and promoting conformational changes associated with transport [42]. We identified the correlation between E320 protonation and substrate Cl^−^ coordination through cryo-EM structure analysis and MD simulations. E320 protonation correlates with stable Cl^−^ binding under simulation conditions. Similar findings were reported in recent research, which also supports the protonation of E320 increase Cl^−^ binding affinity. E320Q mutants, which mimic the protonated state of E320, exhibited decreased currents relative to WT in response to external acidification under high external sulfate conditions. This suggests that cycling of E320 protonation is indispensable for KBAT Cl^−^ channel activity [43]. Located in TM8, E320 can be protonated under pH 8.0 sample solutions, thereby disrupting its interaction with S363 and rendering S363 available for chloride coordination. In addition, given that the equivalent position of E320 is occupied by hydrophobic residues in other paralogs, the correlation between E320 protonation and substrate Cl^−^ binding is special in KBAT (Tables S2 and S3, Figure 3A). The exact and subtle regulation of Cl transport activity in KBAT by E320 protonation still needs physiological evidence, as the complex and dynamic in vivo environments certainly cannot be represented by simple and theoretical simulation conditions.

Substrate Cl^−^ resides in the positively-charged canonical binding pocket of Core domain. The Core domain moves relative to the Gate domain, like an elevator, to submit the substrate to either side of the membrane. This movement comprises both horizontal and vertical orientations. Concomitantly, the crossed topology of TM12–TM5 and TM3–TM10 allows the alternative access to be attained with only a minimal displacement (Figure 4F).

Diseased-related mutations reported in SLC26A2, DRA, Pendrin, Prestin, SLC26A6, SLC26A9, and KBAT are mapped to a structure model of human KBAT in this study [44,45]. Many disease mutations converge to the reported ion binding sites, such as α2 in STAS, TM14, and TM5 in Gate, and TM3 and TM10 in Core [35,38,46] (Figure S2E,F). This underlines that ion recognition and binding capability are fundamental for SLC26 function and regulation.

## 5. Conclusions

Human KBAT plays an essential role in maintaining chloride and sulfate homeostasis. In this study, we elucidated the chloride recognition mechanism of human KBAT, which is markedly influenced by the protonation state of residue E320. Furthermore, quantitative MD contact analysis delineated the interaction rearrangements between the IN and OUT conformations, supporting that KBAT adopts the conserved elevator transport model shared by SLC26 members. Collectively, these findings provide a comprehensive understanding of the distinctive chloride-recognition mechanism in KBAT, paving the way for the rational design of inhibitors aimed at treating pathological neuronal swelling.

## Figures and Tables

**Figure 1:**
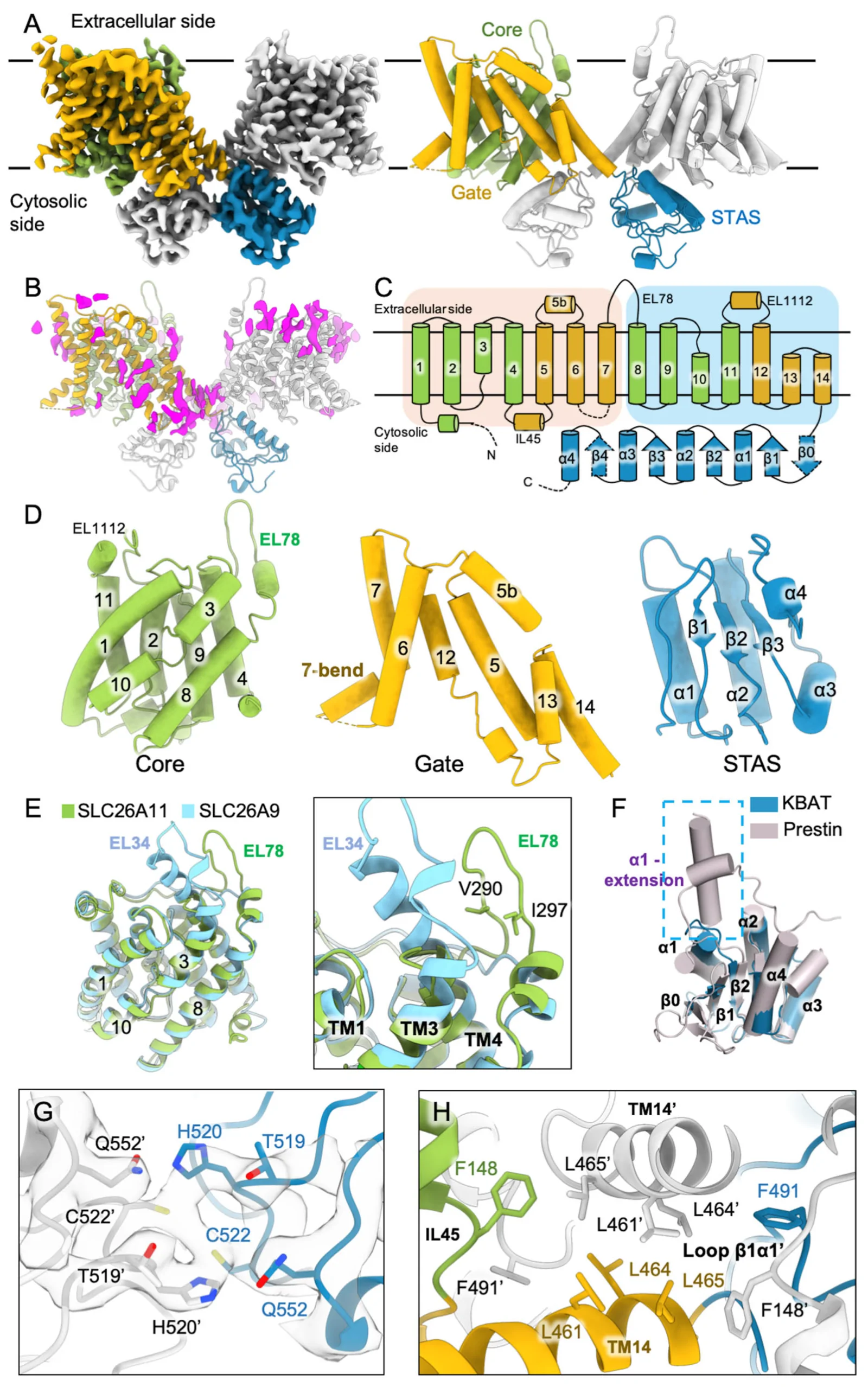
Structure features of human KBAT. (**A**) Cryo-EM map density and overall structure of KBAT are colored by different domains. STAS domains are colored in blue, Core domains in green, and Gate domains in yellow. (**B**) Lipid-like molecules are colored in magenta and presented around the peripheral region of TMD in KBAT. (**C**) A scheme of the secondary structures within KBAT protomer. TM1-7 and TM8-14 form a pseudosymmetry arrangement. (**D**) Zoom-in views of Core, Gate, and STAS domain are shown, respectively. The numbers of major secondary structures are labeled. (**E**) Compared with SLC26A9 (light blue), the KBAT Core domain (green) exhibits shortened EL34 but elongated EL78. V290 and I297 stabilized the EL78 structure. (**F**) Structure alignment of STAS domains of KBAT and Prestin (PDB ID: 7LGU). The shortened loop between α1 and β2 in KBAT is highlighted by blue dashed rectangle. (**G**) Dimerization interface in STAS domain, where H520 and C522 constitute an interaction site. Cryo-EM density in this site is represented by transparent surface. (**H**) Dimerization interface in the neighborhood of TM14 was contributed by some hydrophobic residues in TM14, intracellular loop between TM4 and TM5 (IL45), and loop between β1-α1 of STAS (Loop β1α1).

**Figure 2:**
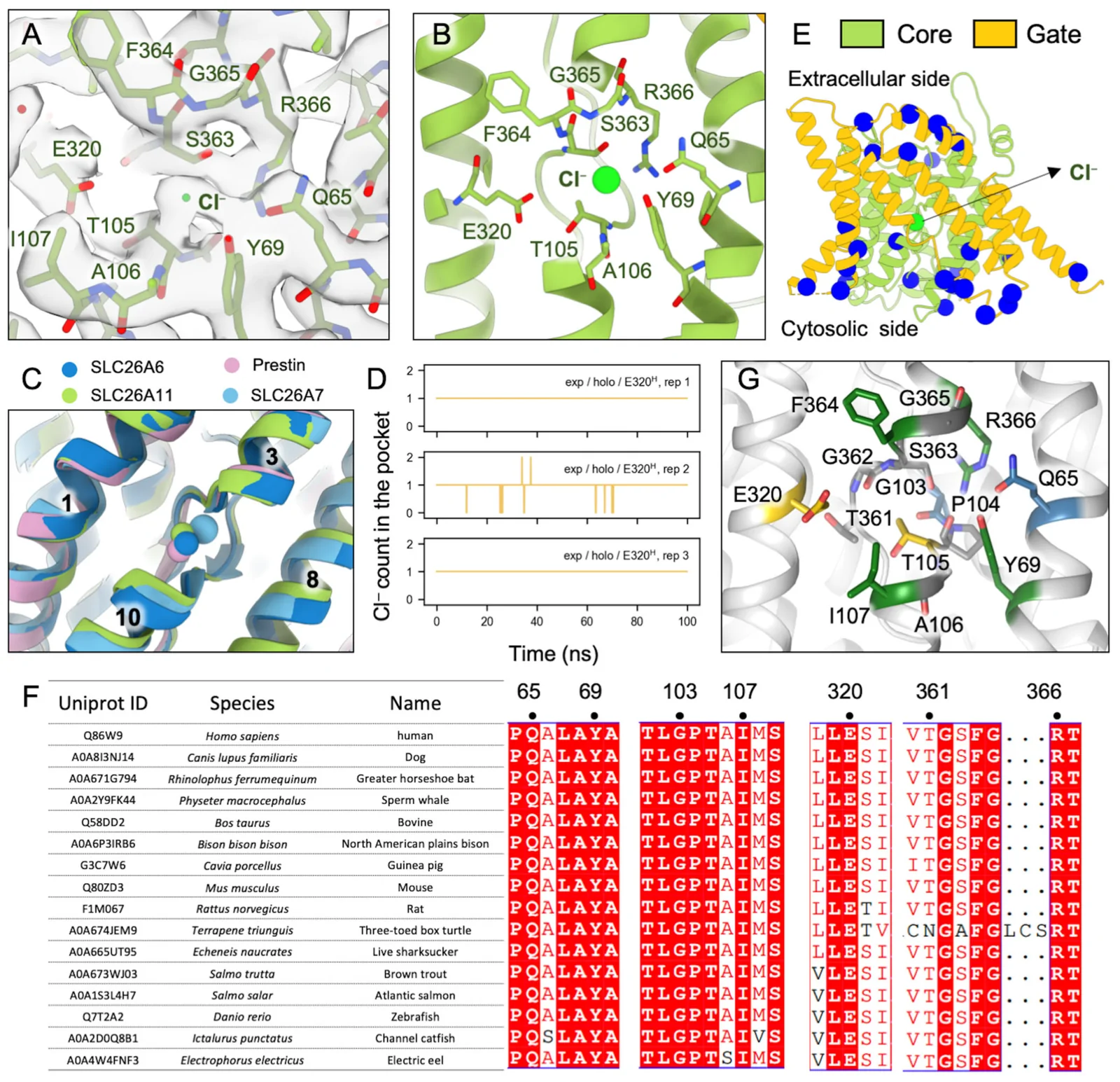
Substrate Cl^−^ binding in human KBAT. (**A**) Cryo-EM density of Cl^−^ binding site are shown in transparent surface. The local resolution can well support the model building and coordination analysis. (**B**) The Cl^−^ is coordinated by main-chain amide groups from T105, A106, F364, G365, and side-chains of S363, Q65, and Y69. E320 does not participate in Cl^−^ coordination directly. (**C**) Structure alignment of halide ion binding in canonical substrate binding pockets of SLC26 experimental structures. Structures of SLC26A6 (PDB: 8OPQ), Prestin (PDB:7LGU), and SLC26A7 (PDB: 9IKV) are used. Halide ions are presented by spheres. (**D**) Number of chloride ions in the binding pocket (defined as within 4 Å of the pocket center) during 100 ns MD simulations, initiated from the experimental structure. “exp/holo/E320^H^” indicates that the initial model used in the simulation is the Cl^−^-bound experimental structure with E320 protonated. (**E**) The Cα of lysine, histidine, and arginine is represented by blue spheres in the TMD of KBAT protomer. (**F**) Sequence alignment of KBAT substrate binding pocket among different orthologues. (**G**) The sequence conservation of substrate binding pocket of SLC26s is represented by different colors. Blue-colored residues are strictly conserved. Green-color represents that KBAT, and other paralogues share the same chemical features. Gray-colored residues are not conserved among paralogues. Yellow-colored sites are special in KBAT. The color pattern is also applied in Table S2.

**Figure 3:**
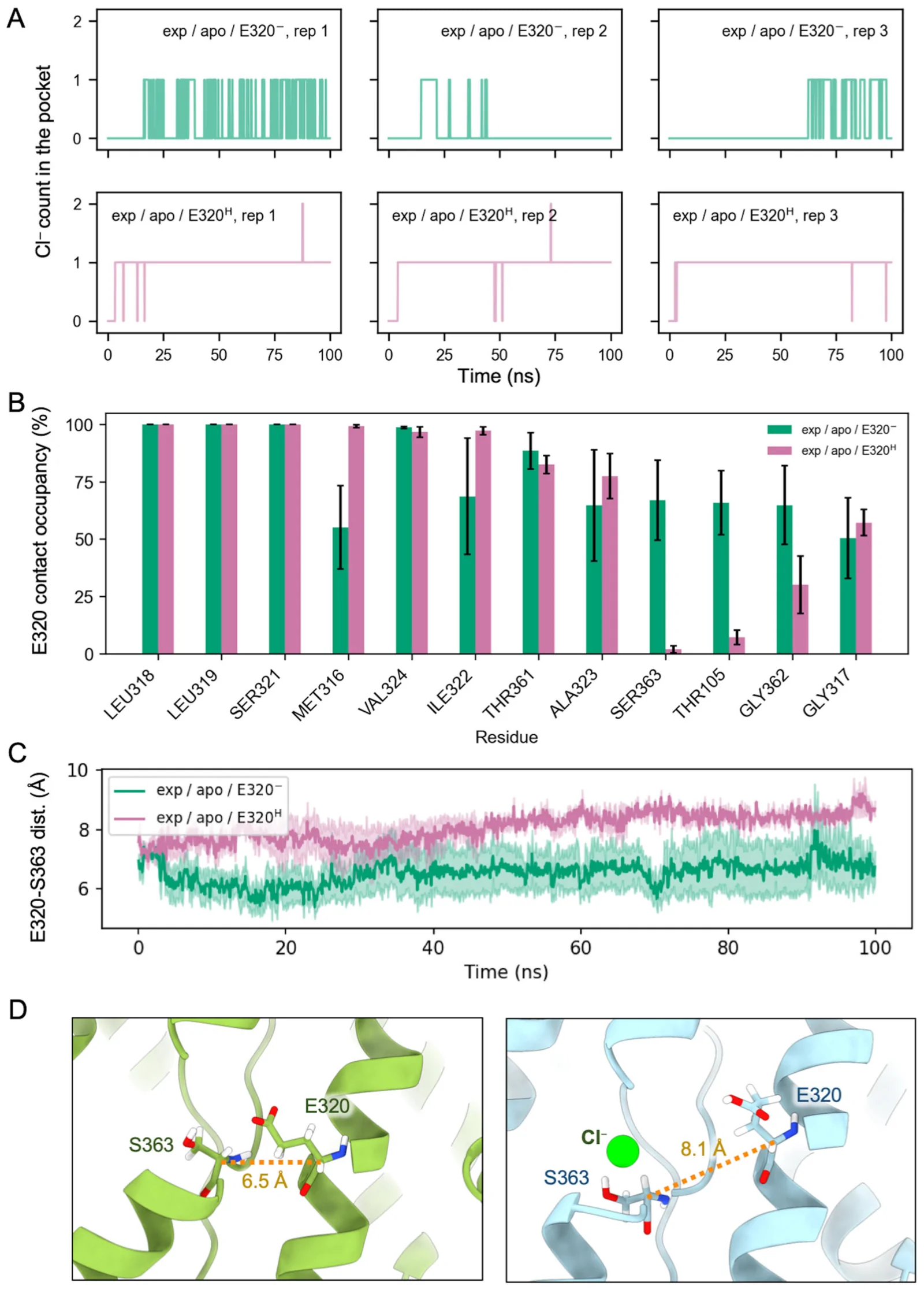
The correlation of E320 protonation and substrate Cl^−^ binding. (**A**) Cl^−^ ion count in the pocket over 100 ns simulations starting from experimental structure in apo with E320 charged (‘exp/apo/E320^−^’) or protonated (‘exp/apo/E320^H^’). In each condition, the simulation was repeated three times. (**B**) The contact occupancy of E320 with other residues in MD simulations. (**C**) The Cα–Cα distance between E320 and S363 during the simulation. The line and the corresponding shade area around each line represent the mean value and SEM. (**D**) A representative conformational snapshot at 50 ns from replica 1 of the simulations with charged E320 (green color) and protonated E320 (light blue color).

**Figure 4:**
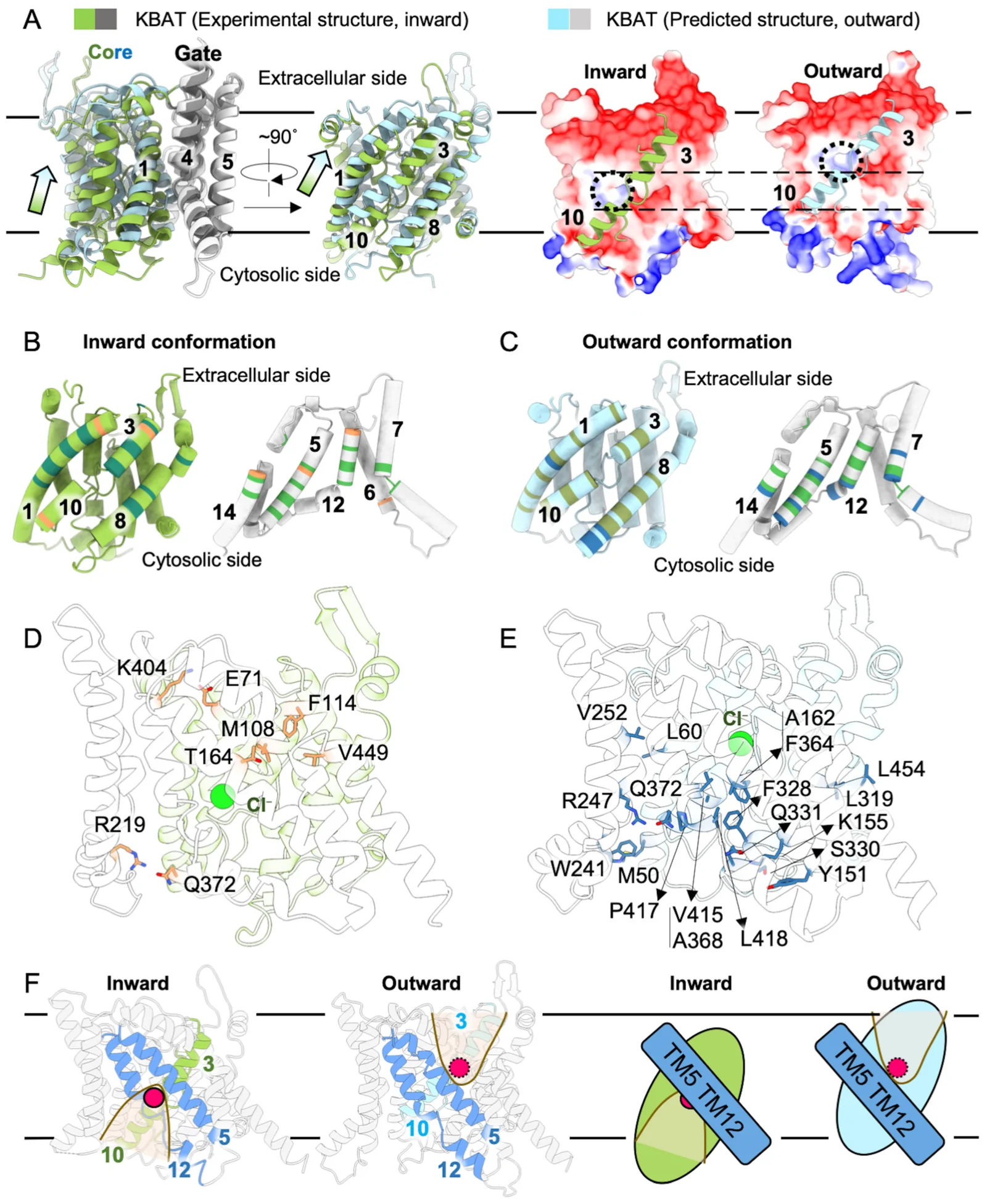
Conformational change in the transport process of KBAT. (**A**) Structure alignment of KBAT experimental structure (inward conformation, colored in green) and AF3 predicted structure (outward conformation, colored in light blue). When conformation changes from inward to outward, obvious displacement of TM3 and TM10 is detected. Substrate binding site (labeled by the dotted circle) is positively charged and moves toward the extracellular side with TM3 and TM10. (**B**) The Core and Gate domains of inward KBAT are shown separately. Core domain is colored in green, and Gate domain is colored in light gray. The orange sites harbor the residues forming interaction solely in IN states. The green sites are residues changing their interaction pairs from IN to OUT conformations. (**C**) same as (**B**), but for outward KBAT. The deep blue sites are residues forming interactions solely in OUT states. (**D**) The interactions solely present in IN conformation are shown in sticks and colored in orange. They are located mostly in the extracellular side of the substrate Cl^−^, which is presented by a green sphere. (**E**) The interactions solely present in OUT conformation are shown in sticks and colored in deep blue. They are located mostly in the cytosolic side of the substrate Cl^−^. (**F**) The cross-arranged pairing of TM5 with TM12 (blue colored) and TM3 with TM10 (green in inward, light blue in outward) allows the Core domain to undergo the limited movement requisite for achieving alternating substrate access to the membrane, a feature that enables the substrate to be presented sequentially to either side of the lipid bilayer. Hot-pink circle represents the substrates.

## Data Availability

The cryo-EM maps for human KBAT have been deposited in the Electron Microscopy Data Bank under the accession codes EMD-67114. The atomic coordinates have been deposited in the Protein Data Bank, under the accession code PDB: 9XQF. They are publicly available as of the date of publication.

## Supplementary Materials

The following supporting information can be downloaded at https://www.mdpi.com/article/10.3390/biom16101384/s1, Figure S1: Data processing of human KBAT; Figure S2: Conformational change in KBAT and disease mutation mapping; Figure S3: Dynamics of pocket Cl^−^ binding as a function of E320 protonation state; Figure S4: Core–Gate domain interactions and chloride ion permeation pathways; Table S1: Statistics of cryo-EM data collection and model-building; Table S2: Sequence alignment of key residues in substrate binding; Table S3: Contact occupancy of a residue in Cl^−^ binding; Table S4: Interaction pairs in different conformations.

## Abbreviations

The following abbreviations are used in this manuscript:

AF3	AlphaFold 3
ATP	Adenosine triphosphate
Cryo-EM	Cryogenic electron microscopy
DDM	N-dodecyl-β-D-maltopyranoside
GDN	Glyco-diosgenin
KBAT	Kidney and brain anion transporter
MD	Molecular dynamic simulations
SLC	Solute carrier family proteins
STAS	Sulfate transporter anti-sigma
TM	Transmembrane
TMD	Transmembrane domain
