# Supplementary material for: Structure Basis for Chloride Recognition and Transport by Human Kidney Brain Anion Transporter (KBAT)
Source: Biomolecules. 2026 Sep 23;16(10):1384. doi: 10.3390/biom16101384 (PMC13702157; doi:10.3390/biom16101384)
Supplement: Supplementary file 1 [file biomolecules-16-01384-s001.zip › biomolecules-4459015 Supplementary new10.5.pdf]

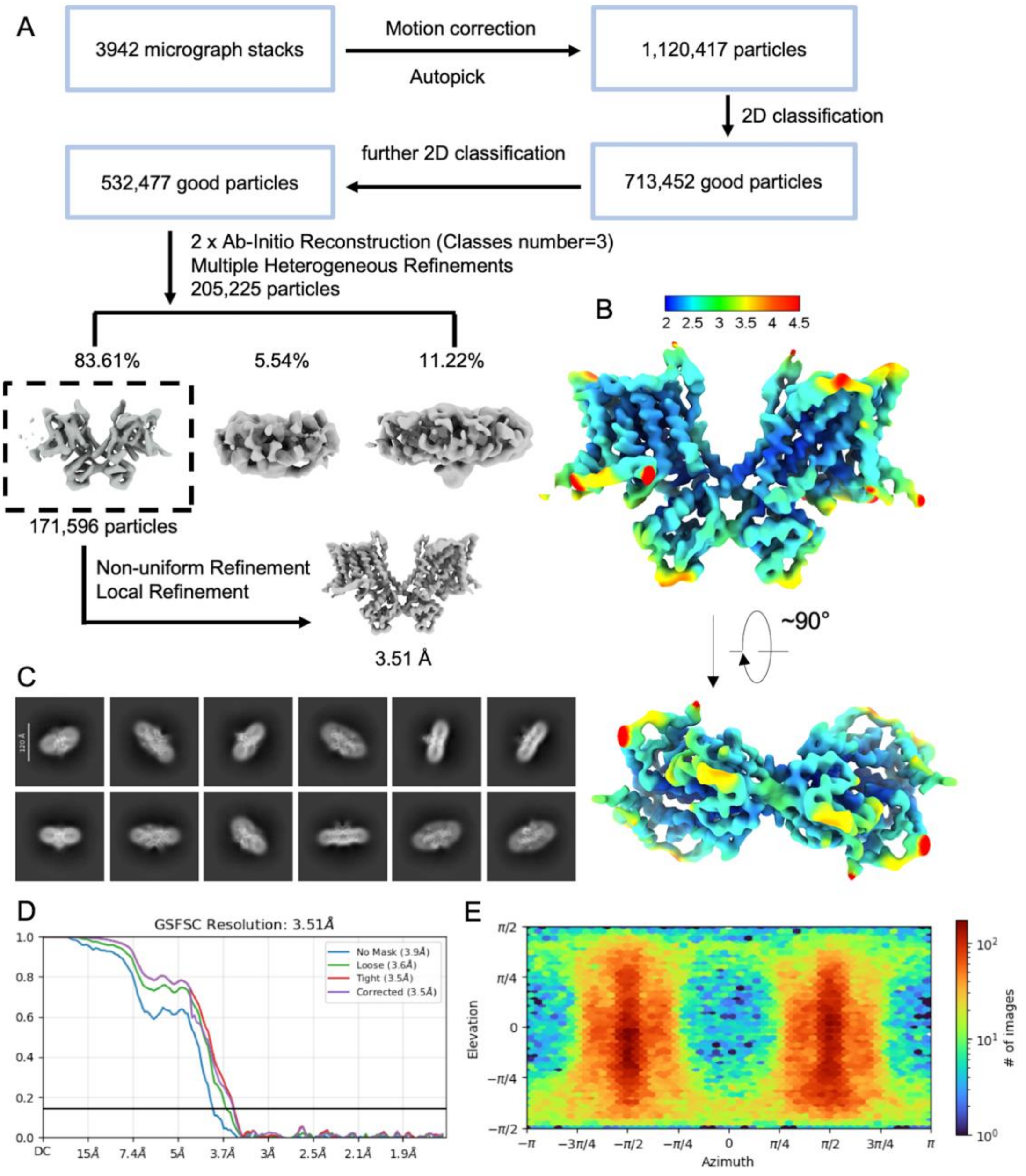

Figure S1. Data processing of human KBAT. (A) Data processing workflow of KBAT. The representative 2D average results are shown in a small panel. (B) Local resolution presentation of the final EM map is colored according to the resolution. (C) Representative 2D classification results in the data processing. (D) Gold-standard Fourier shell correlation (FSC) curve of the cryo-EM map. (E) Angular distribution scheme of the final map.

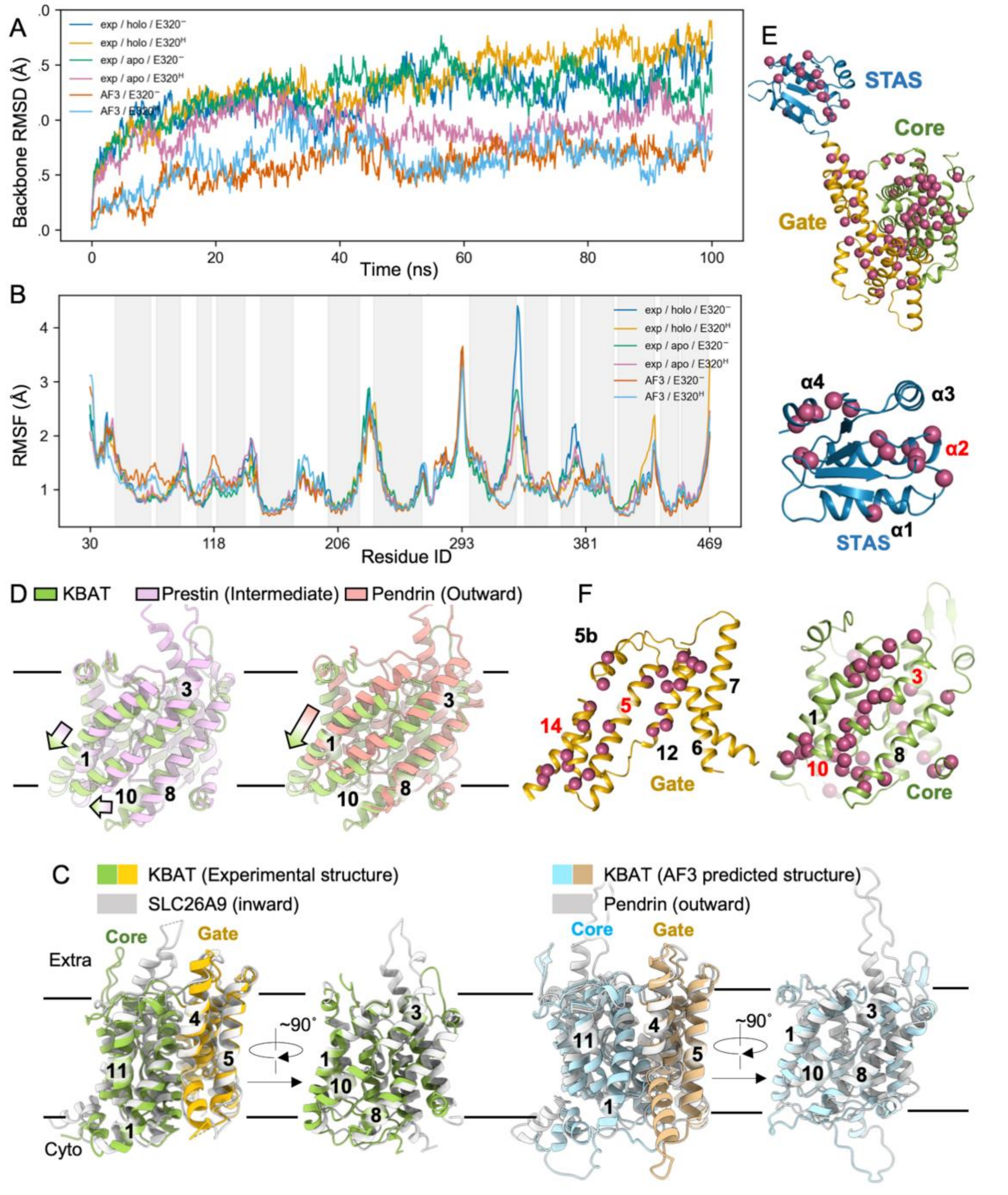

Figure S2. Conformational change in KBAT and disease mutation mapping. (A) Backbone RMSD calculation results for SLC26A11 experimental structure (exp) or AlphaFold 3 predicted structure (AF3) with Cl^- bound (holo)/without Cl^- bound (apo) under condition of E320 charged ($E320^-$) or protonated ($E320^H$) states. (B) Per residue RMSF was calculated and depictured, which reflects residue-level fluctuations. (C) experimental or AF3 predicted structures of KBAT are superposed to SLC26A9 or Pendrin

respectively. The RMSD between experimental KBAT and SLC26A9 (inward) is about 1.089 Å. The RMSD between AF3 predicted KBAT and Pendrin (outward) is about 1.301 Å. (D) Structure alignment between experimental structure of KBAT and Prestin (Intermediate) and Pendrin (Outward) respectively. Substantial displacement of Core domain is shown by colored arrows. (E) Disease related mutations in SLC26s are mapped to KBAT overall and zoom in STAS domain. (F) same as (E), but for Gate and Core domains.

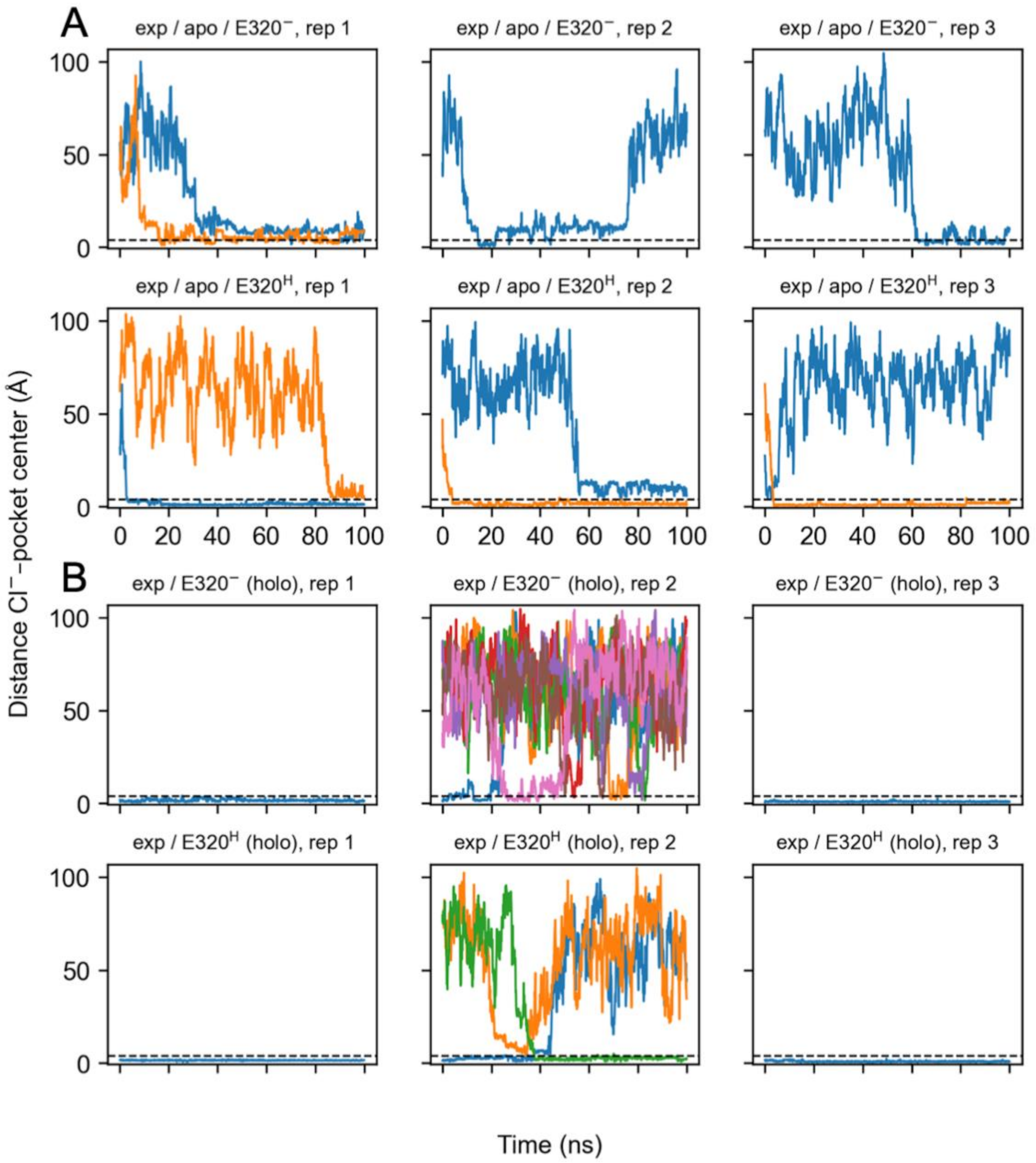

Figure S3. Dynamics of pocket Cl^- binding as a function of E320 protonation state. (A) Per-ion distance between Cl^- and the pocket center over 100 ns MD simulations initialized from apo states. Each row corresponds to one condition ($E320^-$/$E320^H$) and each column to an independent replica; each colored line traces an individual Cl^- ion that entered the pocket (defined as coming within 4 Å of the pocket center). (B) same as (A), but for Cl^- bound holo states.

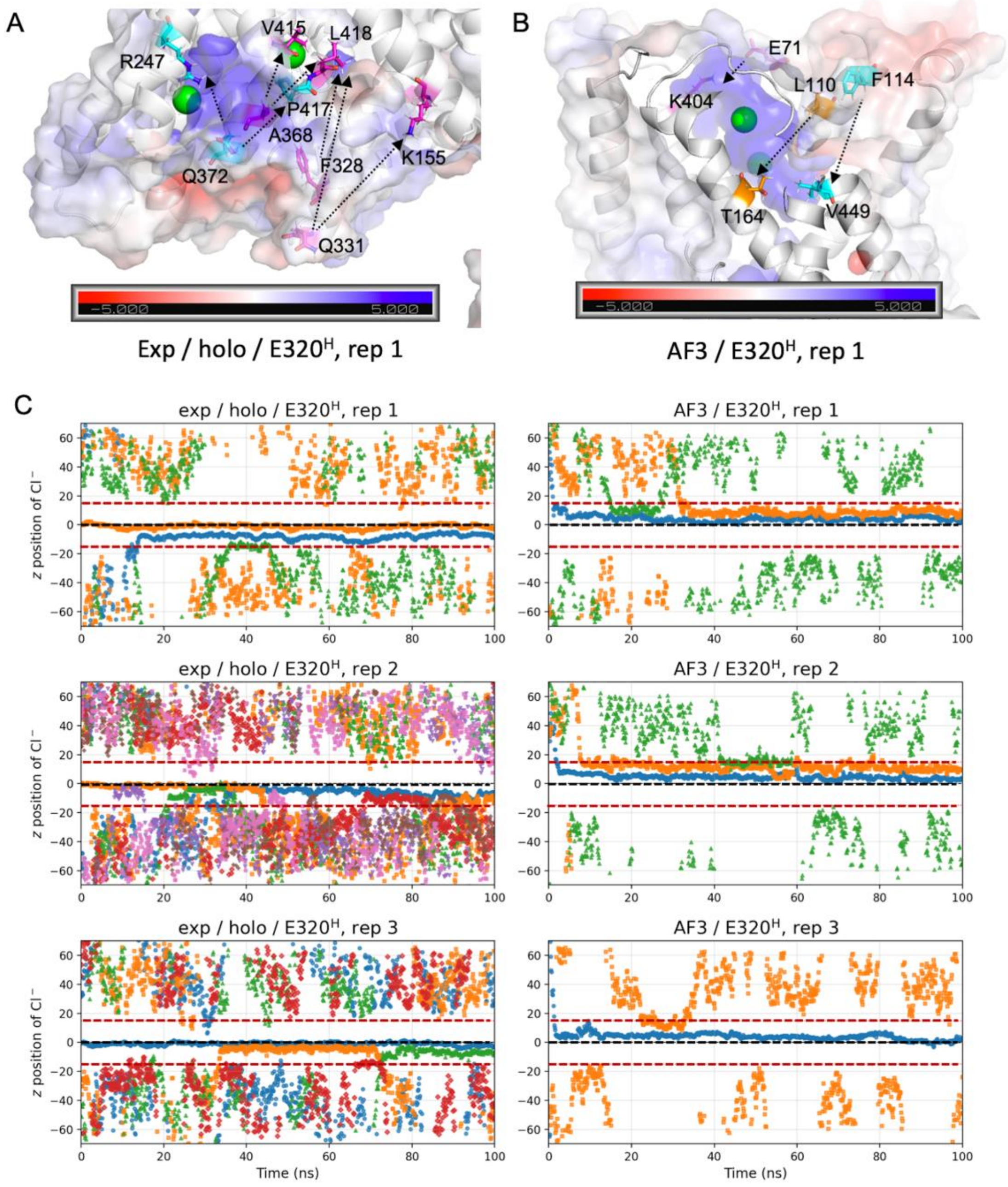

Figure S4. Core–Gate domain interactions and chloride ion permeation pathways. (A) Residues bordering the positively charged chloride-binding vestibule in the final frame of the IN simulation (replica 1) that form exclusive contacts in the OUT state. Arrows indicate contacts from Core domain residues to their contact partners in the Gate domain in another state. The green dot refers to chloride ions. (B) Residues bordering the positively charged chloride-binding vestibule in the final frame of the OUT

simulation (replica 1) that form exclusive contacts in the IN state. (C) Diffusion trajectories of bulk Cl^- ions along the membrane normal (z-axis) relative to the protein center across independent simulation replicas. Each row corresponds to the initial conformation, each column represents an independent simulation replica, and colored solid lines trace individual Cl^- ions that enter the transport vestibule (within 15 Å of the protein center along the z-axis, indicated by the dashed orange line).

Table S1. Statistics of cryo-EM data collection and model-building.

	**SLC2611**<br>PDB-9XQF<br>EMD-67114
**Data collection and Processing**	
Microscope	FEI Titan Krios
Voltage (kV)	300
Camera	K3
Magnification	105,000
Pixel size at detector (Å/pixel)	0.827
Total electron exposure (e-/Å^2)	50
Exposure rate (e-/pixel/sec)	20
Number of frames collected during exposure	32
Defocus range (μm)	-1.5~-0.8
Automation software	EPU
Energy filter slit width (eV)	20
Micrographs collected (no.)	3,942
Total extracted particles (no.)	1,120,417
**Reconstruction**	
Refined particles (no.)	532,477
Final particles (no.)	171,596
Point-group	C2

Resolution (global, Å)	3.51
FSC 0.5 (unmasked/masked)	4.16/4.11
FSC 0.143 (unmasked/masked)	3.52/3.51
Resolution range (local, Å)	2.0-4.5
Map sharpening B-factor ($Å^2$)	
Map sharpening method	CryoSPARC
**Model composition**	
Protein (residues)	1084
**Model refinement**	
Refinement package	Phenix 1.20.1
- real or reciprocal space	Real space
-resolution cutoff	0.5
**Model-Map scores**	
-CC_volume/mask	0.78/0.81
***B* factors ($Å^2$)**	
Protein residues	51.51
**R.m.s deviations from ideal values**	
Bonds length (Å)	0.003
Bonds Angle (°)	0.680
**Validation**	
MolProbity score	2.02
CaBLAM outliers (%)	3.5
Clashscore	11.03
Poor rotamers (%)	0.90
C-beta deviations (%)	0.00
EMRinger score	1.73
**Ramachandran plot**	

Preferred (%)	92.57
Allowed (%)	6.88
Outlier (%)	0.56

Table S2. Sequence alignment of key residues in substrate binding.

	65	69	103	104	105	106	107	320	361	362	363	364	365	366
SLC26A11	Q	Y	G	P	T	A	I	E	T	G	S	F	G	R
SLC26A1	Q	Y	G	I	F	S	L	F	S	A	A	L	A	K
SLC26A2	Q	Y	G	I	F	G	V	I	S	A	A	L	A	K
SLC26A3	Q	F	G	P	F	P	I	V	S	T	A	L	S	R
SLC26A4	Q	Y	G	P	F	P	V	I	T	T	A	L	S	R
SLC26A5	Q	F	G	P	F	A	V	V	S	C	S	L	S	R
SLC26A6	Q	Y	G	T	F	A	V	I	S	C	S	M	S	R
SLC26A7	Q	F	G	T	F	A	L	A	A	A	A	M	G	R
SLC26A8	Q	L	G	S	F	F	L	L	T	G	A	I	A	R
SLC26A9	Q	F	G	T	F	A	V	I	C	C	A	L	S	V

The low sequence conservation renders the direct sequence alignment of SLC26s difficult. Rather, the sites are aligned based on structure superposition. The color pattern is the same as Figure 2F.

Table S3. Contact occupancy of a residue in Cl^- binding.

Residue ID	Residue name	exp / holo / $E320^-$	exp / holo / $E320^H$	exp / apo / $E320^H$
69	TYR	**66.5%**	**93.1%**	**80.3%**
366	ARG	**61.8%**	**79.2%**	**54.3%**
363	SER	**66.0%**	**77.0%**	**88.0%**
105	THR	**34.1%**	**72.4%**	**39.6%**
365	GLY	1.6%	**40.7%**	6.6%
364	PHE	0.6%	**36.7%**	6.3%
106	ALA	2.9%	**29.2%**	11.8%
65	GLN	**11.3%**	**12.1%**	3.6%
104	PRO	9.2%	**11.2%**	8.4%

Bold values indicate occupancy greater than 0.10.

Table S4. Interaction pairs in different conformations.

Interactions in IN only			Pairs in IN	Pairs in OUT
Gate residues	interaction partners in IN Core	interaction partners in OUT Core		
THR164	LEU110(52%)		1	0
ARG219	GLN372(58%)		1	0
LYS404	GLU71(99%)		1	0
VAL449	PHE114(77%)		1	0
Interactions in OUT only				
Gate residues	interaction partners in IN Core	interaction partners in OUT Core		
TYR151		SER330(50%)	0	1
LYS155		GLN331(61%)	0	1
ALA162		PHE364(79%)	0	1
TRP241		MET50(68%)	0	1
ARG247		GLN372(91%)	0	1
VAL252		LEU60(44%)	0	1
VAL415		ALA368(52%)	0	1
PRO417		GLN372(65%)	0	1
LEU418		PHE328(84%); ALA368(63%); GLN331(48%)	0	3
LEU454		LEU319(47%)	0	1
Interactions switches during conformational change between IN and OUT				
Gate residues	interaction partners in IN Core	interaction partners in OUT Core		
ILE154	LEU319(68%); GLU320(58%); MET316(55%)	ALA327(85%); LYS326(73%); ALA323(52%)	3	3
PHE157	ILE107(76%);	ALA323(90%);	3	2

	MET316(58%); LEU111(50%)	VAL324(64%)		
THR158	GLU320(56%); ILE107(46%)	ALA327(97%); PHE364(78%)	2	2
ALA161	ILE107(77%); LEU110(74%)	PHE364(92%)	2	1
ILE165	ALA106(86%); LEU110(59%)	PHE364(44%)	2	1
ARG181	HSD118(57%)	VAL72(60%); LEU110(52%)	1	2
THR245	SER57(75%); THR61(40%)	VAL53(51%); MET50(41%)	2	2
ALA246	LEU60(47%)	VAL53(78%)	1	1
ASN248	PRO64(54%); GLN65(54%); THR61(53%)	SER373(79%); SER57(56%); VAL369(45%)	3	3
ALA249	PRO64(43%)	SER57(93%)	1	1
LEU407	ALA68(52%)	THR61(62%); GLN65(52%)	1	2
VAL410	GLN65(45%)	VAL369(49%)	1	1
ILE411	ALA68(98%); TYR69(65%)	VAL369(46%)	2	1
ALA414	GLN65(58%); TYR69(54%)	ALA368(94%); VAL369(91%); GLN372(82%)	2	3
GLN450	TYR115(91%); ALA308(76%)	MET316(90%); GLU320(75%); ILE107(64%); LEU111(63%)	2	4
ILE453	MET316(60%)	LEU319(65%); ALA323(56%)	1	2
